# Ventilation

**Published:** 1890-01

**Authors:** 


### VENTILATION.

At this time of the year the air is frequently shut out to keep out the cold, and many suffer from the ill effects of an insufficient supply of oxygen, and the breathing of air charged with carbonic acid and other deleterious substances thrown off by exhalation. The evidences of bad ventilation may not be decidedly marked, but the silent and insidious injury to health goes on. A family can be comfortable with less heat and more fresh air than is generally supposed, and in rooms heated by furnace or stoves and lighted by gas too much care regarding ventilation cannot be exercised. Equally important with pure air in living apartments is sunshine. It carries with it radiance and cheer and vigor and good health. It is a purifier, warding off cold, moisture, gloom, depression, and disease. It should be admitted to every apartment of the house, and made welcome at all times. It is a strong preventive to the disorders that visit shaded and musty places. It brings health and happiness that cannot be obtained from any other source. It is nature's own health-giving agent, and nothing can be substituted for it. It has no artificial counterpart. It does not only touch the physical body, but it reaches the mind and soul, and purifies the whole existence of man. It may fade a carpet or upholstery, but it will bring color to the cheek, light to the eye, and elasticity to the step. The closed and shaded window may throw a richness of color upon the room, but it will bring paleness and feebleness to the occupants. This health agent is free to all, easily obtained, and one of the most economic health preservers we have, and ready to impart its efficacy at the rising of the curtain.—*Sanitary News.*

